# Association between sedentary behavior and risk of cognitive decline or mild cognitive impairment among the elderly: a systematic review and meta-analysis

**DOI:** 10.3389/fnins.2023.1221990

**Published:** 2023-08-04

**Authors:** Xiao-ye Cai, Guo-ping Qian, Feng Wang, Ming-yang Zhang, Ying-juan Da, Jing-hong Liang

**Affiliations:** ^1^Department of Physical Education, Shanghai Normal University Tianhua College, Shanghai, China; ^2^Faculty of Physical Culture, Gdansk University of Physical Education and Sport, Gdańsk, Poland; ^3^Department of Physical Education, Shanghai Ocean University, Shanghai, China; ^4^Department of Physical Education, Chengdu Sport University, Chengdu, China; ^5^Department of Maternal and Child Health, School of Public Health, Sun Yat-sen University, Guangzhou, China

**Keywords:** sedentary behavior, cognitive decline, mild cognitive impairment, elderly, meta-analysis

## Abstract

**Background:**

Existing evidence on the association between sedentary behavior (SB) and cognitive function remains inconclusive. Therefore, this study investigated the association between SB and the risk of cognitive decline (CD) or mild cognitive impairment (MCI) in the elderly.

**Methods:**

A comprehensive search was independently conducted by two researchers (XC and GQ) in seven electronic databases, including Medline (via PubMed), China Biology Medicine, Embase, Web of Science, China National Knowledge Infrastructure, Wanfang database, and VIP database for Chinese technical periodicals, covering studies published from the inception of database to June 2023. Studies that investigated the relationship between SB and the risk of CD or MCI in the elderly were included. The quality of the literature was assessed using the Newcastle–Ottawa Scale (NOS) and the Agency for Healthcare Research and Quality (AHRQ) assessment tools. The combined effect size analysis, subgroup analysis, and publication bias assessment were performed using STATA 14.0.

**Results:**

A total of 13 cross-sectional and 6 cohort studies involving 81,791 individuals were included, comprising 17 high-quality studies and 2 medium-quality studies. We found that SB was significantly associated with an increased risk of CD [odds ratio (OR) = 1.69, 95% confidence intervals (CI): 1.47–1.94] or MCI (OR = 1.34, 95% CI: 1.14–1.56) among the elderly. Subgroup analysis stratified according to comorbidity, lifestyle, family structure, publication year, and region showed statistical differences between groups, and the consistency of the results revealed the sources of the heterogeneity.

**Conclusion:**

This meta-analysis showed that SB is positively associated with the risk of CD or MCI in the elderly, providing a higher level of evidence for the promotion of healthy behaviors by clinicians and health policymakers. Due to the number and quality of the included articles, more high-quality longitudinal studies are needed to further confirm our findings.

## Introduction

Cognitive decline (CD) refers to the measurable deterioration observed in various domains of cognitive function, including memory, language, and reasoning (Chun et al., 2021). It represents the preclinical stage of the Alzheimer's disease (AD) continuum, with ~7% of CD per year progressing to mild cognitive impairment (MCI) (Mazzeo et al., 2019). MCI is a state of cognitive impairment between normal aging and dementia (Albert et al., 2011). MCI refers to significant memory deterioration and mild impairment in other domains of cognitive function that do not yet meet the diagnostic criteria for dementia (Langa and Levine, 2014). The incidence of MCI is 20.8%, and ~10–20% of MCI progresses to AD annually (Jia et al., 2020). Cognitive impairment is irreversible, and there are no effective pharmacological or interventional treatments for the condition (Rojas-Fernandez and Cameron, 2012; Yu et al., 2023). Therefore, understanding the risk factors of CD and MCI could enhance the prevention of AD (Petersen et al., 2018).

In 2017, the members of the Sedentary Behavior Research Network (SBRN) defined SB as all activities with ≤ 1.5 metabolic equivalents (METs) of energy expenditure, such as sitting or lying down (Tremblay et al., 2017). SB is common in the elderly and is an independent risk factor for certain diseases (Keadle et al., 2017). Numerous studies have shown that SB is associated with elevated risks of all-cause mortality, cardiovascular mortality, cancer mortality, and the incidence of type 2 diabetes in the elderly population (Bull et al., 2020; Liang et al., 2022). Recently, there has been growing interest in the relationship between SB and cognitive function (Poulin et al., 2016), but issues that have been raised by previous studies remain to be resolved. First, the available evidence remains controversial. For instance, some studies have shown that SB is associated with lower cognitive function and is independently associated with a significantly higher risk of dementia (Falck et al., 2017; Yan et al., 2020). However, two other studies showed that there was no association between SB and cognitive function over time (Kesse-Guyot et al., 2012; Hamer and Stamatakis, 2014). Possible explanations for these differences are that these studies, published over a decade ago, were not appropriately designed, the 2-year follow-up may not have been sufficient to detect longitudinal changes, and SB was self-reported and less objective. Second, previous systematic reviews were based on qualitative studies that had limitations in terms of sample size, population diversity, and the inclusion of individuals with underlying medical conditions (Falck et al., 2017). Thus, further research is needed to improve the quality of evidence and its applicability. Overall, differences in exposures, small sample sizes, short follow-up periods, self-reported SB measures, and uneven quality of review articles often lead to conflicting findings (Hamer and Stamatakis, 2014; Falck et al., 2017). As a result, there is a need to combine and summarize data on the relationship between SB and the risk of CD or MCI to obtain more robust evidence. Thus, we performed a systematic review and meta-analysis of relevant published studies to assess this relationship.

## Methods

This meta-analysis was conducted following the guidelines of Meta-analysis of Observational Studies in Epidemiology (MOOSE) and the Preferred Reporting Items for Systematic Reviews and Meta-analyses (PRISMA) (Stroup et al., 2000; Page et al., 2021). Ethical approval was not required because the study was based on previously published articles. In addition, the authors had no conflicts of interest.

### Search strategies and study selection

The reviewed databases included Medline (via PubMed), China Biology Medicine, Embase, Web of Science, China National Knowledge Infrastructure, Wanfang database, and VIP database for Chinese technical periodicals. They were systematically searched to identify relevant articles published from inception to 11 June 2023. The search was performed using a combination of free words and theme words. Search strategies were developed using Boolean logical operators and truncates. The main search terms included “cognitive dysfunctions,” “mild cognitive impairment,” “cognitive decline,” “cognition,” “sedentary behavior,” “sedentary lifestyle,” “physical inactivity,” and “sedentary time” (see Supplementary material 1 for detailed search strategies). Moreover, articles included in the literature of the relevant systematic review and meta-analysis studies were also searched. This was supplemented by a manual search of conference articles or gray literature to identify references cited in the included literature. When full text was not available, the authors' or corresponding authors' email addresses or other contact details were sought to request full-text access. Two researchers (XC and GQ) independently performed a comprehensive literature search and exported the identified articles to Endnote X9 software (Thompson ISI Research Soft, Philadelphia, USA). After eliminating duplicates manually and electronically with the software, initial screening was performed by reading the titles and abstracts of the articles to exclude irrelevant articles. Next, full-text screening of the articles was conducted, and articles that did not meet the inclusion criteria were removed. In case of discrepancies in the selection of the articles, a senior expert of the research group was consulted to reach a consensus.

### Inclusion and exclusion criteria

Literature inclusion was based on the population, intervention, comparison, outcome, and study design (PICOS) principles of evidence-based medicine (Liberati et al., 2009). The inclusion criteria were as follows: (1) Mean age or age range of the participants ≥ 60 years [The United Nations (UN) defines elderly individuals as those aged 60 years and above (United Nations, 2019)] and CD or MCI patients with undiagnosed dementia. Diagnostic criteria were based on elements from the National Institute of Aging Alzheimer's Association (NIA-AA), the Diagnostic and Statistical Manual of Mental Disorders (DSM), and Petersen's criteria (Montine et al., 2012; Battle, 2013; Petersen et al., 2018); (2) articles were cross-sectional, cohort, and case–control studies, without language restrictions; (3) outcome indicators including CD or MCI, according to the Mini-Mental State Examination (MMSE), the scores ranged from 0 to 30. The boundary values of CD and MCI were divided according to educational level: illiteracy ≤ 17 points, primary school ≤ 20 points, and junior high school and above ≤ 24 points) (Folstein et al., 1975); Montreal Cognitive Assessment (MoCA) (Dong et al., 2012); and Ascertain Dementia 8 (AD 8) (Galvin et al., 2005); and (4) the exposure indicator was SB or physical inactivity. The exclusion criteria were as follows: (1) randomized controlled trials, literature reviews, abstracts, repeatedly published literature, and animal studies literature; (2) studies with incomplete data on outcome indicators; (3) low-quality articles with flaws in the study design; and (4) articles that combined other interventions and did not adjust for confounding factors.

### Data extraction and quality assessment

Two researchers (XC and GQ) extracted data from the included studies separately, and disagreements were resolved by consensus or consultation with a third researcher. Data extracted from the articles included the year of publication, type of study, follow-up period, region, age, sex ratio, sample size, exposure characteristics, and outcome indicators. In addition, when there were several studies in the same article, the data were extracted separately, and in the case of incomplete data, the authors were contacted to request the complete data. Quality assessment of the included studies was conducted using the Newcastle–Ottawa Scale (NOS) and the Agency for Healthcare Research and Quality (AHRQ) (Rostom et al., 2004; Stang, 2010). Specifically, the quality of cohort and case–control studies was assessed using the NOS scale, focusing on three main aspects: sample selection, comparability, and outcomes or exposure, with a total score of 9. A score of one point was assigned for each of the following criteria: consistency of the study with the exposure, selection of the control from the same population, adequate representation of the population, absence of pre-study occurrence of the disease under investigation, and objective ascertainment of exposure. One point each was deducted if the study involved two distinct groups, namely individuals with medical histories requiring further investigation, or recorded the exposure factors through self-reporting. Moreover, two points were assigned in cases of good comparability of studies and correction of important factors. In addition, three points were allocated in cases where outcomes were assessed, follow-up was appropriate, or exposure and response rates were assessed. Points were deducted for failure to mention response rates, missed visits, or short follow-up periods. Scores ranging from 0 to 3 were categorized as low quality, scores from 4 to 6 were considered moderate quality, and scores from 7 to 9 were classified as high quality. For cross-sectional studies, the AHRQ scale was used for quality assessment. It contained 11 items, with a response of “yes,” “no,” and “unclear.” A score ranging from 0 to 3 was classified as low quality, scores from 4 to 7 were considered moderate quality, and scores from 8 to 11 were categorized as high quality. Disagreements on the quality of the literature were resolved by consensus or consultation with another experienced researcher.

### Statistical analyses

Meta-analysis was performed using STATA 14.0 software. The combined odds ratios (OR) and 95% confidence intervals (CI) were used to evaluate the relationship between SB and the risk of CD or MCI in the elderly, with a *P*- value of ≤ 0.05 representing statistically significant differences across the two groups. The existence of heterogeneity among studies was assessed using the *I*^2^ test. If the *P*-value is >0.1 and *I*^2^ ≤ 50%, indicating low heterogeneity among the studies, the fixed effects model was used for the analysis. On the other hand, if the *P*-value is ≤ 0.1 and *I*^2^ > 50%, indicating significant heterogeneity between studies, the random effects model was used for analysis. To address studies with a high level of heterogeneity, subgroup analysis was conducted by dividing the variables into two groups. The sources of heterogeneity were explored based on the following factors: the period of follow-up (≥3 vs. <3 years), region (developed countries vs. developing or undeveloped countries), sample size (≥1,000 vs. <1,000), year of publication (≥2017 vs. <2017), length of SB exposure (≥5 vs. <5 h/day), complications (yes vs. no), lifestyle (adjusted vs. unadjusted smoking, drinking, sleeping, various activity behaviors, and healthy eating), comorbidity (adjusted vs. unadjusted depression, anxiety, obesity, diabetes, stroke, hypertension, medications, medical history, and rheumatic disease), and family structure (adjusted vs. unadjusted marital status, residential status, and living alone). In addition, statistical significance was evaluated by testing for differences across the effect sizes of each subgroup using a 95% CI. Egger's test was used to evaluate publication bias in the included studies, and a *P*-value of >0.05 hypothetically indicated that there was no publication bias.

## Results

### Literature selection

**Figure 1:**
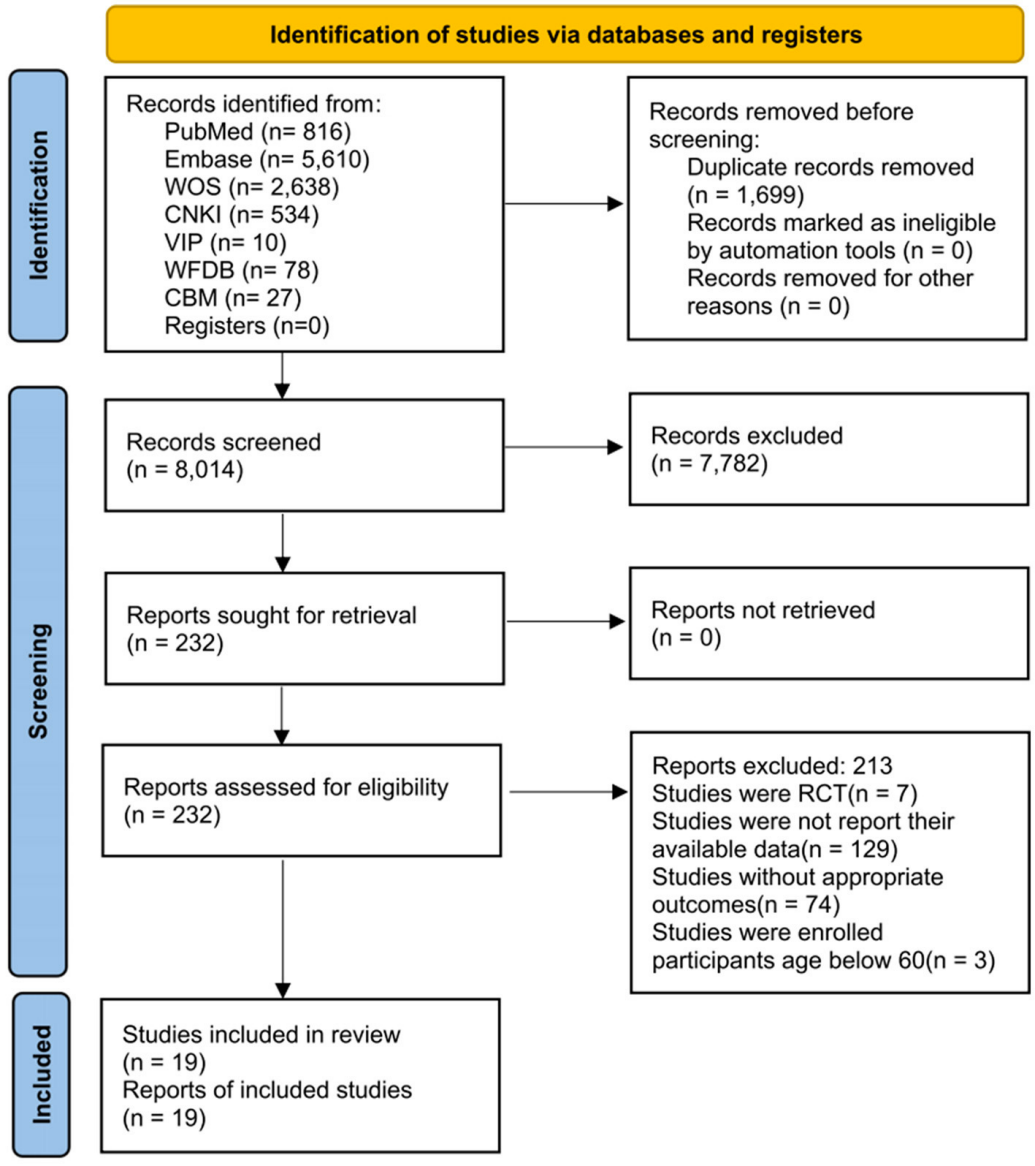
Literature review flowchart. CBM, China biology medicine; CNKI, China national knowledge infrastructure; RCT, randomized controlled trial; VIP, VIP database for Chinese technical periodicals; WFDB, Wanfang database; WOS, Web of Science.

A total of 9,713 relevant articles were retrieved from each database, and 1,699 duplicate articles were removed. After the initial screening of titles and abstracts, 7,782 articles were excluded, and 232 were selected for full-text screening. Furthermore, 213 articles did not meet the criteria and were excluded. Finally, 19 articles (13 cross-sectional and six cohort studies) were included (Ferreira et al., 2010; Dogra and Stathokostas, 2012; Lee et al., 2013; Gillum et al., 2015; Lara et al., 2016; Paulo et al., 2016; Brunner et al., 2017; Gomes et al., 2017; Ku et al., 2017a,b; García-Hermoso et al., 2018; Nemoto et al., 2018; Vancampfort et al., 2018; Martínez-Sanguinetti et al., 2019; Poblete-Valderrama et al., 2019; Jung and Chung, 2020; Cui et al., 2021; Du et al., 2022; Song and Park, 2022). The flow chart illustrating the literature screening process is shown in Figure 1.

### Baseline data of included literature

A total of 19 articles involving 81,791 subjects were included. The included articles originated from 29 countries. In total, 10 of the articles came from developing countries, accounting for 53%, including four articles from China. Articles from developed countries accounted for 47%, with Japan and South Korea each having two articles. Moreover, six of the included articles were cohort studies, with a mean follow-up period of 4.3 years, and the others were cross-sectional studies published between 2012 and 2022. In total, 17 studies reported the sample sizes of the exposure and control groups, and only six reported the proportion of men and women. The method used for cognitive function testing in 11 articles was MMSE, and the testing method in two articles was AD 8. Eight articles analyzed different gradients of sedentary duration or physical inactivity. The detailed basic characteristics of the included studies are shown in Table 1.

**Table 1:** Characteristics of included studies.

**Publications**	**Region**	**Study design**	**Follow-up duration (year)**	**Number of participants**		**Proportion of female (%)**		**Age (mean**±**SD)**		**Exposure group**	**Control group**	**Outcomes**
				**EG**	**CG**	**EG**	**CG**	**EG**	**CG**			
Ku et al. (2017a)	Taiwan, China	CS	2	133	86	NR		74.50 ± 6.10		Medium ST 7–10.99 h/day/High ST 11+ h/day	Low ST < 7 h/day	CD (①)
Ferreira et al. (2010)	Brazil	CS	3	628	232	NR		74.90 ± 6.70		Not favorable behavior trend in PAL + MMSE < 24	Not favorable behavior trend in PAL + MMSE 24+	CD (②)
Lee et al. (2013)	Japan	CS	8	550		47.40%		≥60		Second ST/third ST/highest ST ≤ 1.5 METs-per day (hours)	Lowest ST ≤ 1.5 METs-per day (hours)	CD (②)
Song and Park (2022)	Korea	CS	6	400	450	49.20%	36.60%	71.45 ± 5.32	71.16 ± 4.77	Became inactive/remained inactive	Remained active	CD (②)
				660		58.20%		72.29 ± 5.27				
Gomes et al. (2017)	16 European countries	CSS	1	4,006	1,005	NR		67.80 ± 8.90		Physical inactivity + very good memory	Physical inactivity + poor memory	CD (memory)
Brunner et al. (2017)	UK	CS	5	564	2,837	NR		≥60		Inactivity < 1 h/week moderate and < 1 h/week vigorous PA	Sufficiently active ≥ 2.5 h/week moderate or ≥1 h/week vigorous PA	MCI (②)
Ku et al. (2017b)	Taiwan, China	CS	2	285		NR		≥65		ST ≥ 12 h/day	ST < 8 h/day	MCI (①)
a. Nemoto et al. (2018)	Japan	CSS	1	1,389	952	NR		≥65		Television viewing time 1–2 h/day/2–3 h/day/≥3 h/day	Television viewing time < 1 h/day	MCI
				1,254								
				1,427								
b. Nemoto et al. (2018)	Japan	CSS	1	1,240	1,094	NR		≥65		Time of reading books or newspapers 10–20 min/day/20–30 min/day/≥30 min/day	Time of reading books or newspapers < 10 min/day	MCI
				1,173								
				1,458								
García-Hermoso et al. (2018)	Chile	CSS	2	416/369	573/620	NR		74.13 ± 6.95		Sedentary ≥ 4 h/day/inactive < 600 METs value min/week	Active ≥ 600 METs value min/week/non-sedentary < 4 h/day	MCI (②)
				416/620						Sedentary/active		
Dogra and Stathokostas (2012)	Canada	CSS	1	3,204	4,862	54.40%	56.40%	≥65		Moderately sedentary 2–4 h/day/Least sedentary < 2 h/day	Sedentary > 4 h/day	MCI
				1,412		53.00%					
Vancampfort et al. (2018)	China, Ghana, India, Mexico, Russia, and South Africa	CSS	4	3,304	29,411	NR		62.10 ± 15.60		SB ≥ 8 h/day/SB per 1 h increase	SB < 8 h/day	MCI
Lara et al. (2016)	Spain	CSS	2	1,160	1,385	NR		66.26 ± 0.18		Low PA	Moderate PA	MCI
Cui et al. (2021)	China	CSS	1	358	733	NR		70.40 ± 6.60		SB ≥ 5 h/day	SB < 5 h/day	CD+MCI (②)
Du et al. (2022)	China	CSS	1	139	287	47.40%	46.70%	69.90 ± 5.80		SB ≥ 8 h/day	SB < 8 h/day	MCI (②)
Gillum et al. (2015)	US	CSS	2	194	1,162	NR		≥60		Sitting screen-hours > 3 h/day	Sitting screen-hours-3.1 in others	MCI
Jung and Chung (2020)	Korea	CSS	1	7,888	1,776	59.40%	52.02%	74.08 ± 6.50	72.50 ± 6.14	TV viewing	Not involved in TV viewing	MCI (②)
Martínez-Sanguinetti et al. (2019)	Chile	CSS	2	169	1,215	NR		≥60		High SB ≥ 4 h/day	Low SB < 4 h/day	MCI (②)
Paulo et al. (2016)	Brazil	CSS	1	51	340	64.70%	60.60%	71.07 ± 7.77		Physical inactivity < 150 min of MVPA/week	PA ≥ 150 min of MVPA/week	MCI (②)
Poblete-Valderrama et al. (2019)	Chile	CSS	2	169	1,215	NR		>60		High SB 4–8 h/day/very high SB > 8 h/day	Low SB < 4 h/day	MCI (②)

CD, cognitive decline; CG, control group; CS, cohort study; CSS, cross-sectional study; EG, exposure group; MCI, mild cognitive impairment; METs, metabolic equivalants; MVPA, moderate to vigorous physical activity; NR, no reported; Outcome: AD8 (ascertain dementia8); MMSE (mini-mental state examination); PA, physical activity; PAL, physical activity level; SB, sedentary behavior; SD, standard deviation; ST, sedentary time. In addition, a. Nemoto and b. Nemoto are the same article. The characteristics of different exposure groups in the same article are distinguished by/markers.

### Quality of the included studies

According to the quality evaluation standard, 17 of the articles were high-quality studies, while two were medium-quality studies. One study met all the quality evaluation criteria. Three studies were limited by one factor, and the others were limited by at least two factors, with a research quality score of 6–9. The loss-to-follow-up rate of two articles was >25%, and the follow-up period of three articles was <5 years. All articles did not provide clarity regarding whether the evaluators' factors covered other aspects of the research object. Furthermore, it remains unclear whether follow-up data were available, including information on the expected population with incomplete data or follow-up results. Seven articles explained how missing data were handled in the analysis. The quality assessment of the included studies is specified in Supplementary Tables 1, 2.

### Results of traditional meta-analysis

#### Association between SB and the risk of CD in the elderly

**Figure 2:**
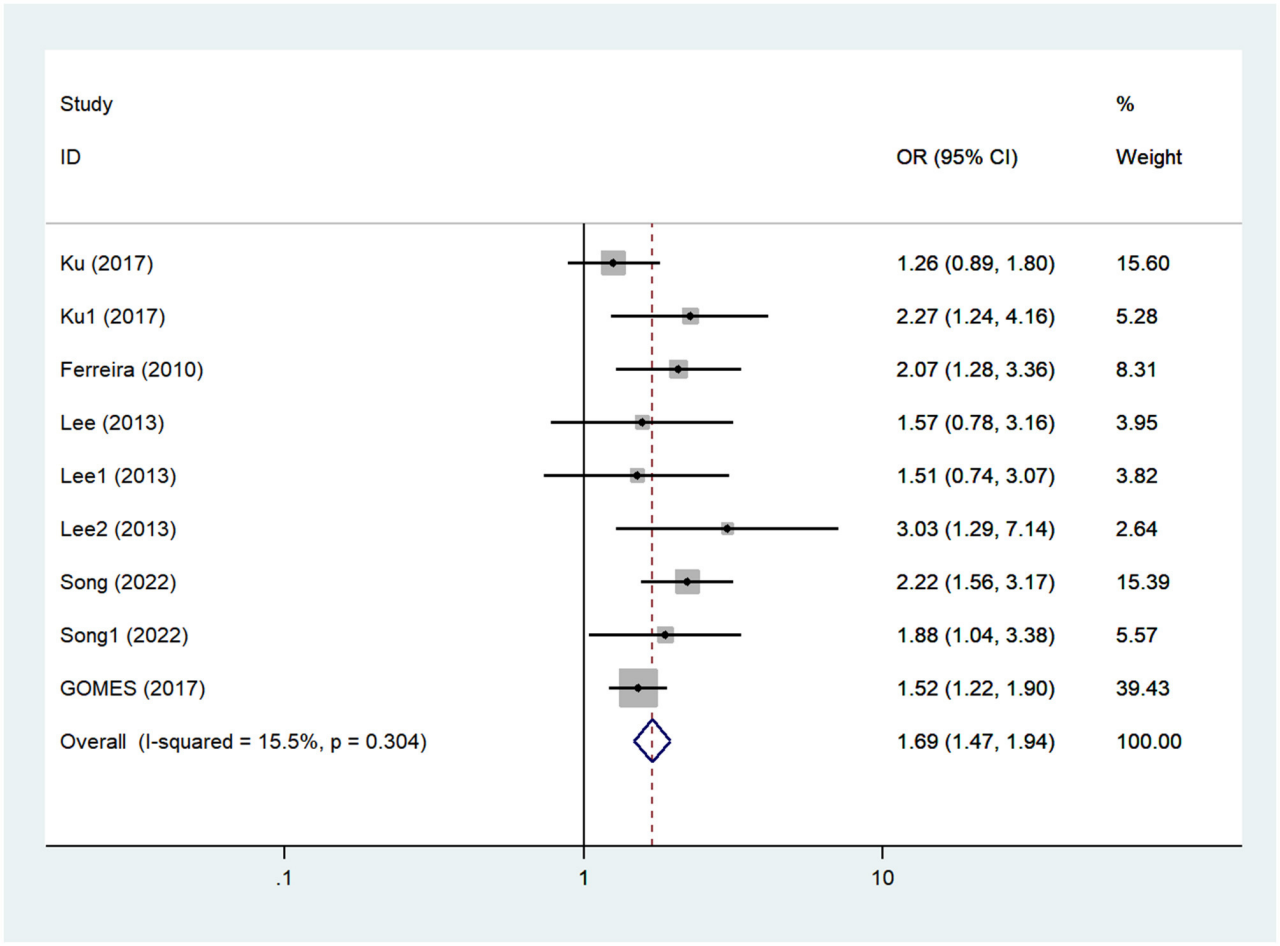
Forest plot of cognitive decline (CD).

Five articles investigated the relationship between SB and the risk of CD in the elderly, with a sample size of 8,439 cases (Ferreira et al., 2010; Lee et al., 2013; Gomes et al., 2017; Ku et al., 2017a; Song and Park, 2022). The heterogeneity test showed that there was no heterogeneity among the studies (*I*^2^ = 15.50%, *P*_heterogeneity_ = 0.30); thus, the fixed effect model was used for meta-analysis. The combined meta-analysis results demonstrated a significant difference between the two groups (OR = 1.69, 95% CI: 1.47–1.94, *P* < 0.01), and long-term SB was found to increase the risk of CD in the elderly compared to the control group, as shown in Figure 2 and Table 2. The funnel diagram was relatively symmetrical, and Egger's test suggested that there was no publication bias within the studies ($P_{\text{Egge}{\text{r}}^{\text{′}}\text{stest}}$ = 0.19), as shown in Supplementary Figures 1, 2.

**Table 2:** Results of traditional meta-analysis and subgroup analyses.

**Meta-analyses outcomes/subgroup**		**Number of studies**	**OR, 95% CI**	**Heterogeneity**		**Effect model**
				***P***	*I*^2^ **(%)**	
**Meta-analysis of the association between SB and risk of CD or MCI in elderly**						
(High vs. Low) SB + CD		5	1.69 (1.47, 1.94)	0.30	15.50	Fixed effect model
(High vs. Low) SB + MCI		14	1.34 (1.14, 1.56)	< 0.10	92.70	Random effects model
**Results of subgroup analyses about SB and the risk of CD in elderly**						
Duration of follow-up	Overall	5	1.69 (1.47, 1.94)	0.30	15.50	Fixed effect model
	≥3 years	3	2.03 (1.63, 2.53)	0.80	0.00	Fixed effect model
	< 3 years	2	1.05 (1.25, 1.79)	0.25	27.40	Fixed effect model
Region	Overall	5	1.69 (1.47, 1.94)	0.30	15.50	Fixed effect model
	Developed countries	3	1.72 (1.46, 2.03)	0.39	2.90	Fixed effect model
	Developing or underdeveloped countries	2	1.61 (1.25, 2.09)	0.12	51.70	Fixed effect model
Publication year	Overall	5	1.69 (1.47, 1.94)	0.30	15.50	Fixed effect model
	≥2017	3	1.64 (1.41, 1.91)	0.15	40.50	Fixed effect model
	< 2017	2	1.93 (1.40, 2.66)	0.58	0.00	Fixed effect model
Total sample size	Overall	5	1.69 (1.47, 1.94)	0.30	15.50	Fixed effect model
	≥1,000	2	1.71 (1.43, 2.04)	0.19	38.80	Fixed effect model
	< 1,000	3	1.67 (1.34, 2.08)	0.28	19.00	Fixed effect model
SB exposure time	Overall	5	1.69 (1.47, 1.94)	0.30	15.50	Fixed effect model
	≥5 h/day	2	1.58 (1.23, 2.02)	0.26	23.10	Fixed effect model
	< 5 h/day	3	1.75 (1.48, 2.07)	0.28	21.20	Fixed effect model
Complications	Overall	5	1.69 (1.47, 1.94)	0.30	15.50	Fixed effect model
	Yes	5	1.69 (1.47, 1.94)	0.30	15.50	Fixed effect model
	No		–	–	–	–
Comorbidities	Overall	5	1.69 (1.47, 1.94)	0.30	15.50	Fixed effect model
	Adjusted	5	1.69 (1.47, 1.94)	0.30	15.50	Fixed effect model
	Unadjusted		–	–	–	–
Lifestyle	Overall	5	1.69 (1.47, 1.94)	0.30	15.50	Fixed effect model
	Adjusted	3	1.78 (1.47, 2.15)	0.26	21.60	Fixed effect model
	Unadjusted	2	1.60 (1.31, 1.96)	0.25	23.10	Fixed effect model
Family structure	Overall	5	1.69 (1.47, 1.94)	0.30	15.50	Fixed effect model
	Adjusted	2	1.76 (1.42, 2.19)	0.12	48.60	Fixed effect model
	Unadjusted	3	1.64 (1.37, 1.97)	0.49	0.00	Fixed effect model
**Results of subgroup analyses about SB and the risk of MCI in elderly**						
Duration of follow-up	Overall	14	1.34 (1.14, 1.56)	0.00	92.70	Random effects model
	≥3 years	2	1.27 (0.99, 1.62)	0.00	85.50	Random effects model
	< 3 years	12	1.41 (1.11, 1.78)	0.00	93.40	Random effects model
Region	Overall	14	1.34 (1.14, 1.56)	0.00	92.70	Random effects model
	Developed countries	6	0.99 (0.79, 1.23)	0.00	92.90	Random effects model
	Developing or Underdeveloped countries	8	2.18 (1.53, 3.09)	0.00	92.10	Random effects model
Publication year	Overall	14	1.34 (1.14, 1.56)	0.00	92.70	Random effects model
	≥2017	9	1.39 (1.14, 1.70)	0.00	94.50	Random effects model
	< 2017	5	1.25 (1.10, 1.42)	0.34	11.60	Random effects model
Total sample size	Overall	14	1.34 (1.14, 1.56)	0.00	92.70	Random effects model
	≥1,000	10	1.14 (0.98, 1.33)	0.00	91.90	Random effects model
	< 1,000	4	2.77 (1.47, 5.21)	0.00	85.00	Random effects model
SB exposure time	Overall	14	1.34 (1.14, 1.56)	0.00	92.70	Random effects model
	≥5 h/day	8	1.42 (1.14, 1.78)	0.00	94.70	Random effects model
	< 5 h/day	6	1.26 (1.16, 1.37)	0.46	0.00	Random effects model
Complications	Overall	14	1.34 (1.14, 1.56)	0.00	92.70	Random effects model
	Yes	13	1.37 (1.15, 1.62)	0.00	93.40	Random effects model
	No	1	1.16 (0.98, 1.38)	0.52	0.00	Random effects model
Comorbidities	Overall	14	1.34 (1.14, 1.56)	0.00	92.70	Random effects model
	Adjusted	12	1.34 (1.12, 1.59)	0.00	93.50	Random effects model
	Unadjusted	2	1.33 (0.97, 1.83)	0.04	68.10	Random effects model
Lifestyle	Overall	14	1.34 (1.14, 1.56)	0.00	92.70	Random effects model
	Adjusted	10	1.32 (1.10, 1.59)	0.00,	94.00	Random effects model
	Unadjusted	4	1.38 (1.08, 1.77)	0.02	63.60	Random effects model
Family structure	Overall	14	1.34 (1.14, 1.56)	0.00	92.70	Random effects model
	Adjusted	7	1.27 (0.96, 1.69)	0.00	94.50	Random effects model
	Unadjusted	7	1.49 (1.24, 1.79)	0.00	83.30	Random effects model

SB, sedentary behavior; CD, cognitive decline; MCI, mild cognitive impairment.

#### Association between SB and the risk of MCI in the elderly

**Figure 3:**
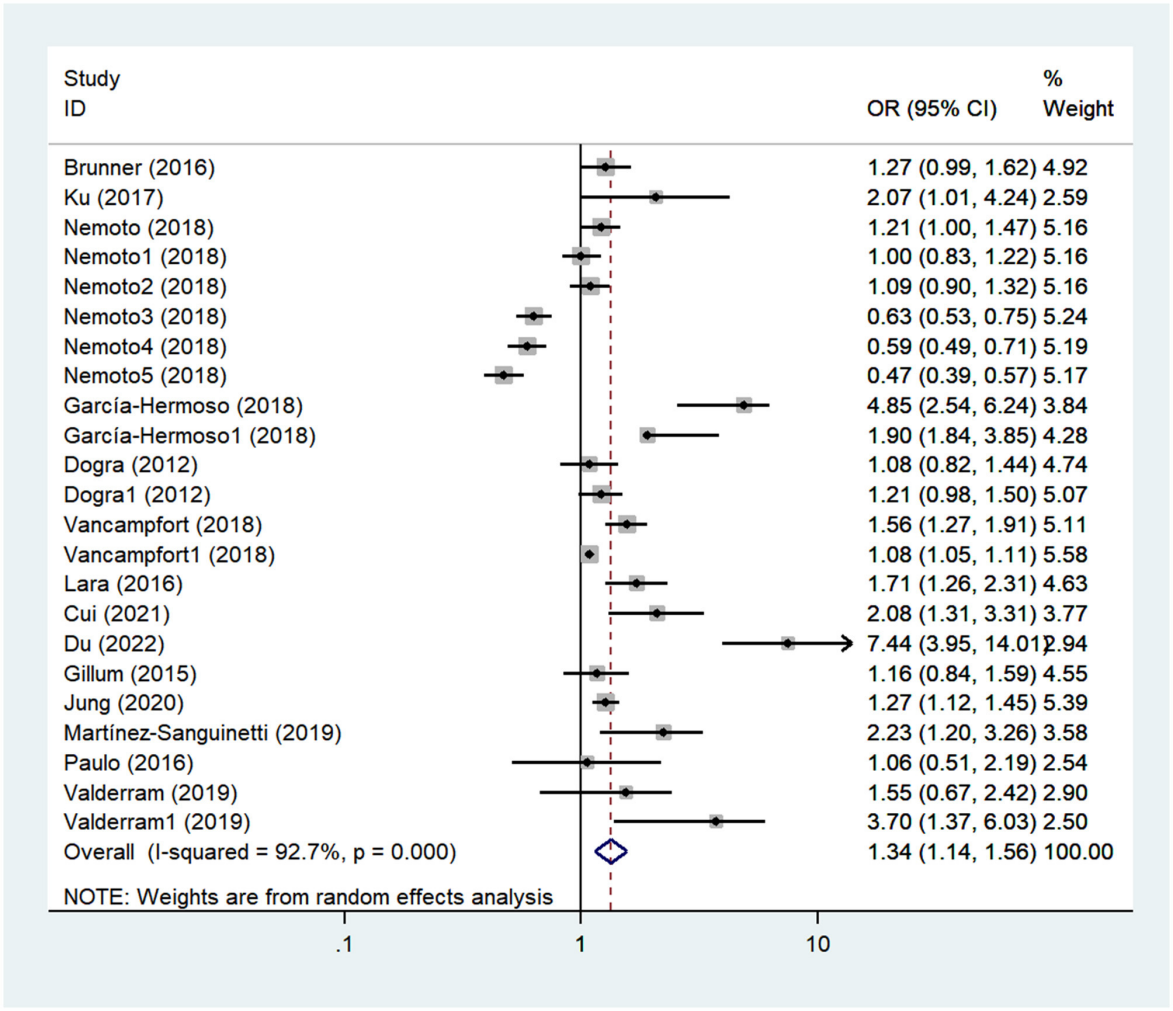
Forest plot of mild cognitive impairment (MCI).

In total, 14 articles investigated the relationship between SB and the risk of MCI in the elderly, with a sample size of 73,352 cases (Dogra and Stathokostas, 2012; Gillum et al., 2015; Lara et al., 2016; Paulo et al., 2016; Brunner et al., 2017; Ku et al., 2017b; García-Hermoso et al., 2018; Nemoto et al., 2018; Vancampfort et al., 2018; Martínez-Sanguinetti et al., 2019; Poblete-Valderrama et al., 2019; Jung and Chung, 2020; Cui et al., 2021; Du et al., 2022). The heterogeneity test showed that the studies were heterogenous (*I*^2^ = 92.70%, *P*_heterogeneity_ < 0.10), and a random effects model was used for the meta-analysis. The combined results showed a significant difference between the two groups (OR = 1.34, 95% CI: 1.14–1.56, *P* < 0.01), and prolonged SB was found to increase the risk of MCI in the elderly compared with control groups, as shown in Figure 3 and Table 2. The funnel diagram was relatively symmetrical, and Egger's test suggested that there was no publication bias within the studies ($P_{\text{Egge}{\text{r}}^{\text{′}}\text{stest}}$ = 0.20), as shown in Supplementary Figures 3, 4.

### Results of subgroup analyses

No subgroup statistical differences were observed in the relationship between SB and the risk of CD across the two groups or were any significant sources of heterogeneity identified. Significant statistical differences were observed in most subgroups regarding the relationship between SB and the risk of MCI. Furthermore, the consistency among these subgroups provided insights into the sources of heterogeneity. Subgroup analysis of SB exposure time showed significantly higher heterogeneity for sedentary time above 5 h/day (OR = 1.42, 95% CI: 1.14–1.78, *P*_heterogeneity_ = 0.00, *I*^2^ = 94.70%, random model) compared to that within 5 h/day (OR = 1.26, 95% CI: 1.16–1.37, *P*_heterogeneity_ = 0.46, *I*^2^ = 0.00%, random model) though no difference was observed between these two subgroups. Detailed results for other subgroup analyses are shown in Table 2.

## Discussion

This study explored the association between SB and the risk of CD or MCI in the elderly from the perspective of evidence-based medicine. We found that elderly individuals with long-term SB were more likely to develop CD or MCI than those who engaged in physical activity (PA) or had short-term SB, and this trend is even more evident in Asian populations.

This study showed that prolonged SB increased the risk of CD in the elderly (OR = 1.69, 95% CI: 1.47–1.94), and there was a significant positive correlation between prolonged SB and increased risk of CD. Related studies have also revealed results consistent with ours (Kesse-Guyot et al., 2014; Falck et al., 2017). In a longitudinal study investigating the association of low PA, SB, smoking, and other lifestyle parameters with cognitive function in 2,430 middle-aged and older adults, Kesse-Guyot et al. (2014) showed that SB was associated with a decline in overall cognitive function in the elderly. In a systematic review of the association between SB and cognitive function, Falck et al. found that SB is not only associated with a higher risk of type 2 diabetes and cardiovascular disease but can also lead to a decline in overall cognitive, memory, and executive function in elderly individuals (Falck et al., 2017). In a cross-sectional study, Coelho et al. (2020) found that the negative association between SB and cognitive function was predominantly significant in the elderly with long-term SB. This could be attributed to the fact that with an increase in the sedentary time of elderly individuals, the body correspondingly induces an increase in white matter hyperintensity volume (WMHV), a decrease in the level of brain-derived neurotrophic factor (BD-NF), and a decrease in the level of medial temporal changes such as the thinning of leaf thickness and abnormal cerebral blood flow, which can lead to a decline of cognitive function in the elderly. In conclusion, the mechanisms through which SB causes CD are complex and multifaceted. Possible explanations for the discrepancy between existing relevant evidence (Kesse-Guyot et al., 2012; Hamer and Stamatakis, 2014) and the result of this article are that the sample size of their study was small and unrepresentative and that the short-term follow-up may not have been sufficient to detect a meaningful association between changes in cognition and SB. Moreover, the level of cognitive stimulation in the elderly also varies across SB measurement modes. In contrast, our study employed a comprehensive search strategy and involved multiple databases, and a complementary search was performed for potential literature such as meeting reports and abstracts. Consequently, the sample size of this study is large enough to provide strong evidence of the association under study. In addition, our results also found that prolonged SB significantly increased the risk of MCI in the elderly (OR = 1.34, 95% CI: 1.14–1.56), and there was a significant positive correlation between prolonged SB and the risk of MCI, consistent with previous studies (Xie et al., 2021; Gafni et al., 2022). In a cross-sectional study, Xie et al. (2021) found a significant relationship between excessive SB and MCI in the elderly. Their findings also revealed that limiting sedentary time is important as elderly patients with MCI tend to have a greater sedentary time, thereby leading to more adverse health outcomes. In another cross-sectional study, Gafni et al. (2022) found that insufficient PA and sitting for at least three-quarters of the day increased the risk of MCI in the elderly. In other words, low PA and prolonged SB adversely affect cognitive function in the elderly (Peng et al., 2022). This could be due to the high prevalence of chronic inflammation and reduced hormone levels associated with older stages of life. Long-term SB aggravates bone loss, reduces the size and quantity of muscle fibers, affects the contraction of skeletal muscles, and accelerates cell aging and brain atrophy, leading to MCI in the elderly. Thus, reducing sedentary time could prevent or retard the development of MCI. Moreover, it is highly recommended that elderly individuals should increase their PA level and improve their sleep quality to enhance their individual cognitive function. Sleep duration, SB, and PA are co-dependent behaviors that constitute the movement/non-movement continuum and together account for the 24-h daily cycle (Zhu et al., 2020). Studies have shown that PA plays a positive role as an effective cognitive intervention in helping to improve cognitive function in older adults with AD or MCI (Liang et al., 2018). A minimum of 150 min of moderate-to-vigorous physical activity (MVPA) per week promotes an increased blood flow to the brain and improves metabolism and cardiovascular health (Liang et al., 2018). Deep sleep plays a crucial role in facilitating the clearance of amyloid-beta (Aβ) in the brain and improving overall sleep quality (Liang et al., 2020). They contribute to reducing the risk of MCI and dementia in the elderly population (Liang et al., 2020). Therefore, we encourage older adults to meet the 24-h healthy movement guidelines (i.e., ≥60 min of MVPA, ≤ 2 h of screentime, and age-appropriate sleep duration) (Zhu et al., 2020) in order to improve their cognitive health outcomes. In addition, we investigated whether there are gender differences in the cognitive impairment caused by SB. Studies have shown that the prevalence of MCI is 1.28 times higher in women than in men (Wang et al., 2020). Men who are engaged in mental work have rich knowledge reserves and strong thinking abilities and are not prone to cognitive impairment (Wang et al., 2020). Therefore, we suggest that elderly individuals should be encouraged to participate in educational activities, such as playing computer games or chess, to exercise their memory and potentially delay cognitive dysfunction.

In the subgroup analysis, we found significant differences between the two groups in terms of lifestyle, comorbidity, and family structure. The reason may be that sedentary elderly individuals live alone for a long time, have reduced daily communication, and find it difficult to overcome loneliness, anxiety, and depression, which could affect their cognitive function. The brain weight of elderly individuals decreases with increasing age, and there is a certain degree of inevitable physiological brain aging (Ma et al., 2019). The emergence of other comorbidities (such as hypertension and diabetes) accelerates the decline and impairment of cognitive function in the elderly. Simultaneously, there were differences in region, publication year, and the duration of SB between the two groups. This is due to the level of regional economic and medical resources, the construction of elderly activity venues, the lengthiness of publication, the short duration of the study, the sample size of the articles, and the quality of the content of the studies. In addition, this study mostly used scales, structured interview questionnaires, and self-reports to measure sedentary behavior, leading to possible bias in the results. Future research should use objective methods to more accurately measure the duration of SB, such as a three-dimensional accelerometer and an inclinometer.

### Strengths and limitations

To the best of our knowledge, this is the first study to examine the relationship between SB and the risk of CD or MCI in the elderly. Given that this area of research is still developing, our study only provides some insight into the relationship between SB and the risk of CD or MCI in the elderly, providing reliable evidence for the development of future public health policies. The articles included in our study were small, and studies were not of high quality, which may have led to some bias in the interpretation of the results. In addition, the definition of “sedentary behavior” was inconsistent, which might induce bias in effect size estimates. For the evaluation of article quality, the existence of subjective judgment errors could lead to judgment bias. In the future, other assessment methods should be considered to minimize these errors to a great extent. When conducting subgroup analysis, some subgroups included a small number of articles and sample sizes, which reduced the reliability of the results. This requires further validation by high-quality and large-sample studies. This study is based on a systematic review and meta-analysis of observational studies. Most of the articles included in the analysis were from cross-sectional studies, limiting the ability to infer causality. In the future, more high-quality randomized controlled trials (RCTs), Mendelian randomization (MR) studies, or basic research would be needed.

## Conclusion

In summary, our study reveals a positive association between SB and the risk of CD or MCI in the elderly. Long-term SB increases the risk of CD or MCI in the elderly. We recommend that the elderly reduce their SB time and increase their level of PA to promote healthy cognitive aging. Considering the quantity and quality of the included articles, our findings need to be interpreted with caution, and more high-quality longitudinal studies are required in the future to further demonstrate the association between SB and the risk of CD or MCI in the elderly.

## Data availability statement

Data generated or analyzed during this study are included in this published article or in the data repositories listed in the references.

## Author contributions

X-yC served as the principal author, had full access to all data in the study, and took responsibility for the accuracy of the data analysis and the integrity of the data. J-hL and FW contributed to the conception and design. M-yZ, G-pQ, and X-yC contributed to data acquisition and interpretation. X-yC and G-pQ contributed to the draft of the manuscript. Y-jD and J-hL revised the article and finally approved the study. All authors contributed to the article and approved the submitted version.

## Supplementary material

Supplementary material 1 Search strategies.

Supplementary Figure 1 Funnel plot of cognitive decline (CD).

Supplementary Figure 2 Egger's test of cognitive decline (CD).

Supplementary Figure 3 Funnel plot of mild cognitive impairment (MCI).

Supplementary Figure 4 Egger's test of mild cognitive impairment (MCI).

Supplementary Table 1 Quality assessment for inclusion in cohort studies.

Supplementary Table 2 Quality assessment for inclusion in cross-sectional studies.
