# Supplementary material for: Association between sedentary behavior and risk of cognitive decline or mild cognitive impairment among the elderly: a systematic review and meta-analysis
Source: Front Neurosci. 2023 Aug 4;17:1221990. doi: 10.3389/fnins.2023.1221990 (PMC10436513; doi:10.3389/fnins.2023.1221990)
Supplement: Supplementary material 1 — Search strategies. [file Table_1.DOCX]

# Search strategy

**PubMed: 816**

#11 #3 AND #10 816

#10 "#6 OR #9 327598

#9 #7 OR #8 120631

#8 "cognitive dysfunction*"[Title/Abstract] OR "dysfunction* cognitive"[Title/Abstract] OR "cognitive impairment*"[Title/Abstract] OR "impairment* cognitive"[Title/Abstract] OR "mild cognitive impairment*"[Title/Abstract] OR "cognitive impairment* mild"[Title/Abstract] OR "impairment* mild cognitive"[Title/Abstract] OR "mild neurocognitive disorder*"[Title/Abstract] OR "disorder* mild neurocognitive"[Title/Abstract] OR "neurocognitive disorder* mild"[Title/Abstract] OR "cognitive decline*"[Title/Abstract] OR "decline* cognitive"[Title/Abstract] OR "deterioration* mental"[Title/Abstract] OR "mental deterioration*"[Title/Abstract] 113518

#7 "Cognitive Dysfunction"[MeSH Terms] 32508

#6 #4 OR #5 239099

#5 "Cognition*"[Title/Abstract] OR "cognitive function*"[Title/Abstract] OR "function* cognitive"[Title/Abstract] 72057

#4 "Cognition"[MeSH Terms] 190726

#3 #1 OR #2 27769

#2 "behavior* sedentary"[Title/Abstract] OR "sedentary lifestyle"[Title/Abstract] OR "lifestyle sedentary"[Title/Abstract] OR "physical inactivity"[Title/Abstract] OR "inactivity physical"[Title/Abstract] OR "lack of physical activity"[Title/Abstract] OR "sedentary time*"[Title/Abstract] OR "time sedentary"[Title/Abstract] 19905

#1 "Sedentary Behavior"[MeSH Terms] 13034

**Web of Science: 2638**

#5 #1 AND #4 2638

#4 #2 OR #3 480567

#3 TS=(Cognitive Dysfunction* OR Dysfunction*, Cognitive OR Cognitive Impairment* OR Impairment*, Cognitive OR Mild Cognitive Impairment* OR Cognitive Impairment*, Mild OR Impairment*, Mild Cognitive OR Mild Neurocognitive Disorder* OR Disorder*, Mild Neurocognitive OR Neurocognitive Disorder*, Mild OR Cognitive Decline* OR Decline*, Cognitive OR Deterioration*, Mental OR Mental Deterioration*) 234946

#2 TS=(Cognition* OR Cognitive Function* OR Function*, Cognitive) 355620

#1 TS=(Sedentary Behavior* OR Behavior*, Sedentary OR Sedentary Lifestyle OR Lifestyle, Sedentary OR Physical Inactivity OR Inactivity, Physical OR Lack of Physical Activity OR Sedentary Time* OR Time*, Sedentary) 61126

**Embase: 5610**

#8 #2 AND #7 5610

#7 #4 OR #6 3302336

#6 'cognitive defect'/exp OR 'cognitive dysfunction*':ab,ti OR 'dysfunction* cognitive':ab,ti OR 'cognitive impairment*':ab,ti OR 'impairment* cognitive':ab,ti OR 'mild cognitive impairment*':ab,ti OR 'cognitive impairment* mild':ab,ti OR 'impairment* mild cognitive':ab,ti OR 'mild neurocognitive disorder*':ab,ti OR 'disorder* mild neurocognitive':ab,ti OR 'neurocognitive disorder* mild':ab,ti OR 'cognitive decline*':ab,ti OR 'decline* cognitive':ab,ti OR 'mental deterioration*':ab,ti OR 'deterioration* mental':ab,ti OR 'Cognitive Dysfunction*':ab,ti 612458

#5 'cognitive defect'/exp 581447

#4 'cognition'/exp OR 'Cognition*':ab,ti OR 'cognitive function*':ab,ti OR 'function* cognitive':ab,ti 2898511

#3 'cognition'/exp 2877966

#2 'sedentary lifestyle'/exp OR 'behavior* sedentary':ab,ti OR 'sedentary behavior*':ab,ti OR 'sedentary lifestyle':ab,ti OR 'lifestyle sedentary':ab,ti OR 'physical inactivity':ab,ti OR 'inactivity physical':ab,ti OR 'lack of physical activity':ab,ti OR 'sedentary times*':ab,ti OR 'time* sedentary':ab,ti 40399

#1 'sedentary lifestyle'/exp 18600

**Chinese National Knowledge Infrastructure: 534**

#2 主题=# 1 和 "轻度认知障碍"或"轻度神经认知障碍"或"认知减退"或"精神衰退"或"认知损害"或"神经行为障碍"或"认知功能下降"或"精神退化"或"认知能力下降"或"认知功能障碍"或"认知"或"认知功能"或"认知能力" 534

#1 主题=''久坐行为"或"静态行为"或"久坐不动"或"久坐少动"或"静坐不动"或"静坐少动"或"缺乏体育活动"或"身体不活动"或"静态生活方式"或"久坐时间"或"静态少动行为"或"久坐生活方式" 9250

**Wanfang Database: 78**

#1 主题="久坐行为"或"静态行为"或"久坐不动"或"久坐少动"或"静坐不动"或"静坐少动"或"缺乏体育活动"或"身体不活动"或"静态生活方式"或"久坐时间"或"静态少动行为"或"久坐生活方式" 和 "认知"或"认知功能"或"认知能力" 或 "轻度认知障碍"或"轻度神经认知障碍"或"认知减退"或"精神衰退"或"认知损害"或"神经行为障碍"或"认知功能下降"或"精神退化"或"认知能力下降"或"认知功能障碍" 78

**VIP database for chinese technical periodicals: 10**

#1 题名或关键词="久坐行为"+"静态行为"+"久坐不动"+"久坐少动"+"静坐不动"+"静坐少动"+"缺乏体育活动"+"身体不活动"+"静态生活方式"+"久坐时间"+"静态少动行为"+"久坐生活方式"和"认知"+"再认"+认知功能"+"认知能力"或"轻度认知障碍"+"轻度神经认知障碍"+"认知减退"+"精神衰退"+"认知损害"+"神经行为障碍"+"认知功能下降"+"精神退化"+"认知能力下降"+"认知功能障碍" 10

**China Biology Medicine: 27**

#11 #3 和 #10 27

#10 "#6 或 #9 157217

#9 #7 或 #8 24470

#8 常用字段="轻度认知障碍"或"轻度神经认知障碍"或"认知减退"或"精神衰退"或"认知损害"或"神经行为障碍"或"认知功能下降"或"精神退化"或"认知能力下降"或"认知功能障碍" 24470

#7 "认知功能障碍"[不加权:扩展] 11963

#6 #4 或 #5 156918

#5 常用字段="认知"或"认知功能"或"认知能力" 156918

#4 "认知"[不加权:扩展] 58169

#3 #1 或 #2 560

#2 常用字段="久坐行为"或"静态行为"或"久坐不动"或"久坐少动"或"静坐不动"或"静坐少动"或"缺乏体育活动"或"身体不活动"或"静态生活方式"或"久坐时间"或"静态少动行为"或"久坐生活方式" 560

#1 "久坐生活方式"[不加权:扩展] 18
