# Supplementary figures and images for: Association between sedentary behavior and risk of cognitive decline or mild cognitive impairment among the elderly: a systematic review and meta-analysis
Source: Front Neurosci. 2023 Aug 4;17:1221990. doi: 10.3389/fnins.2023.1221990 (PMC10436513; doi:10.3389/fnins.2023.1221990)

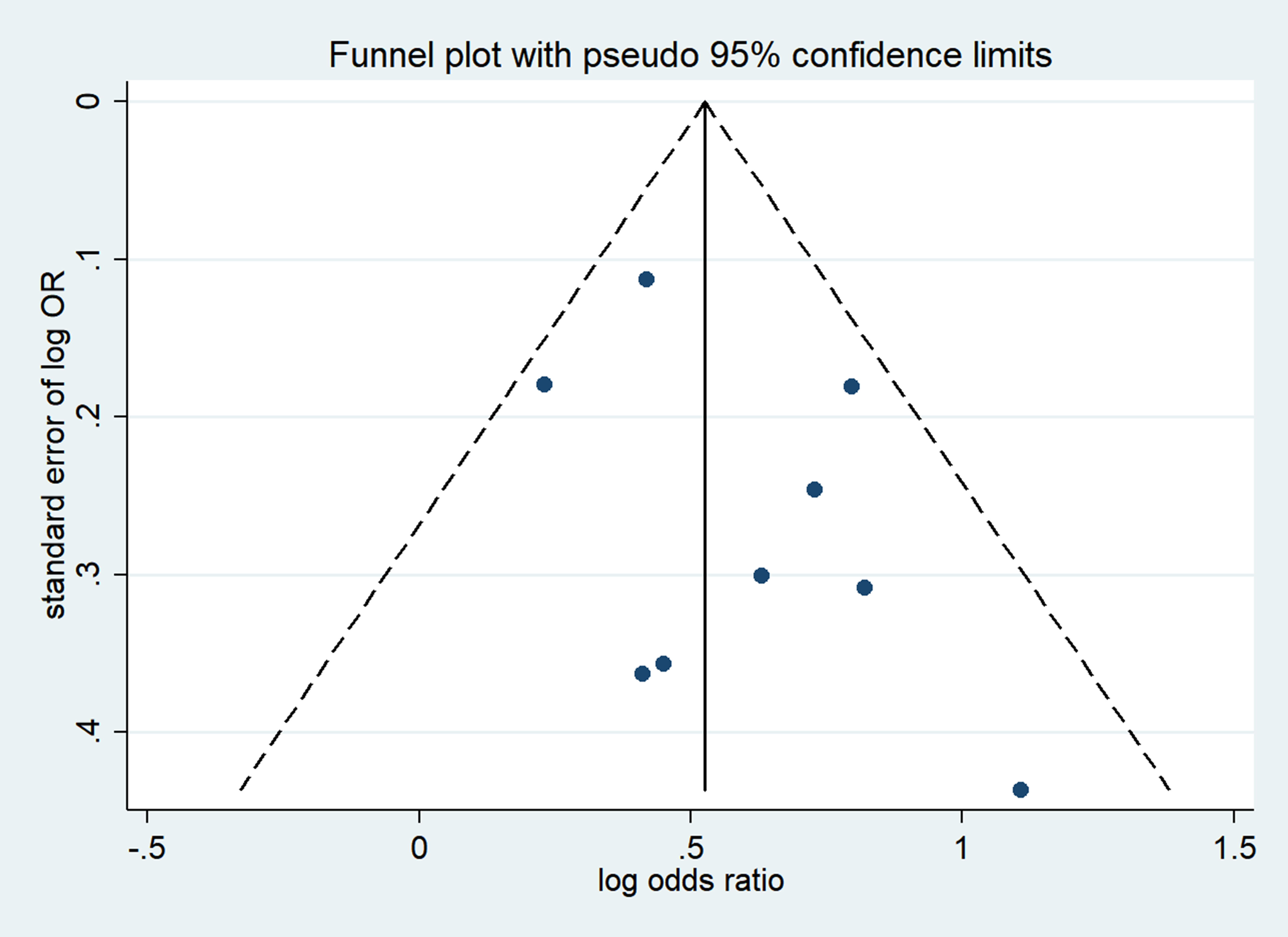

Supplement: Supplementary Figure 1 — Funnel plot of cognitive decline (CD). [file Image_1.TIF]

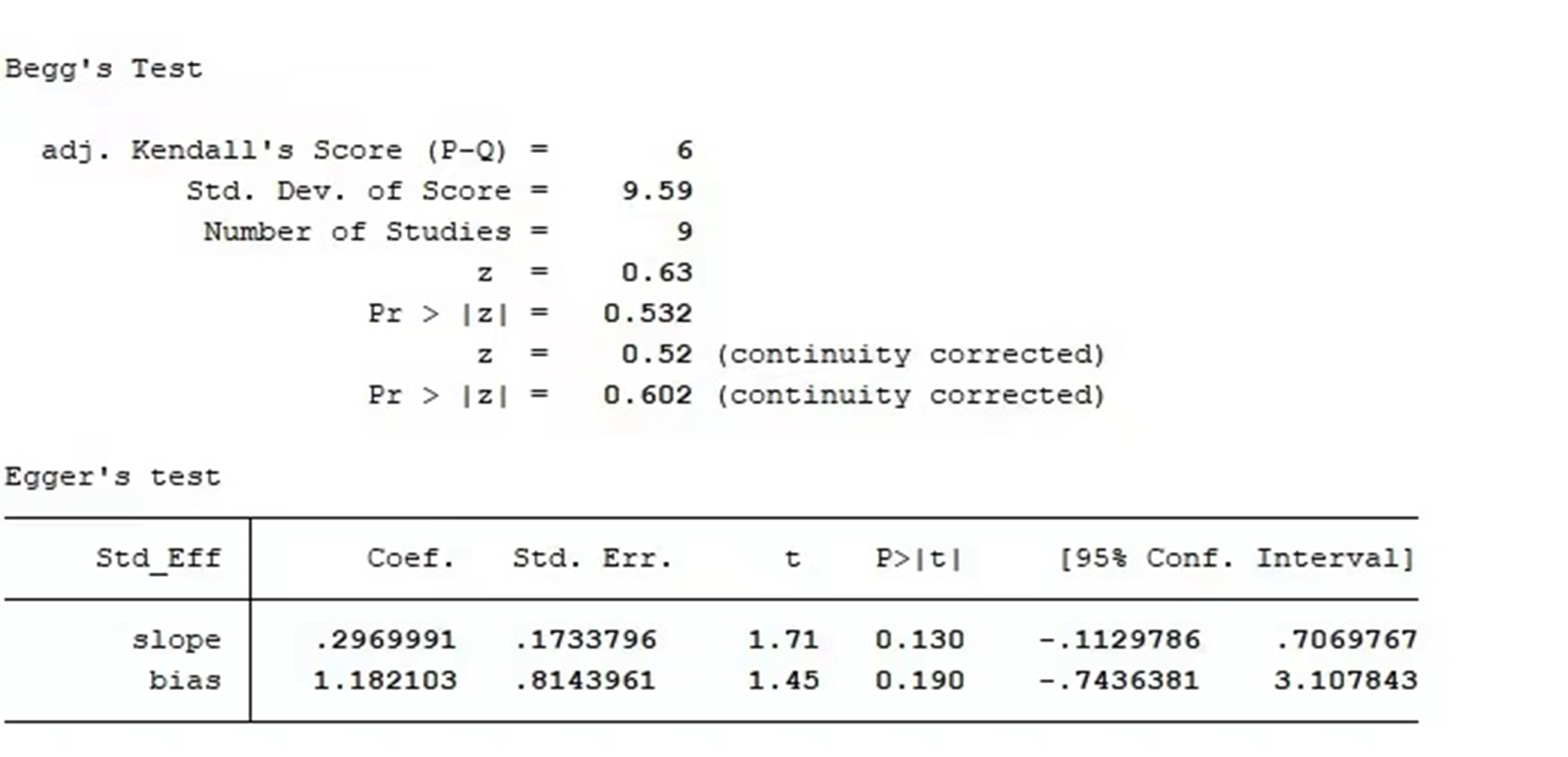

Supplement: Supplementary Figure 2 — Egger's test of cognitive decline (CD). [file Image_2.TIF]

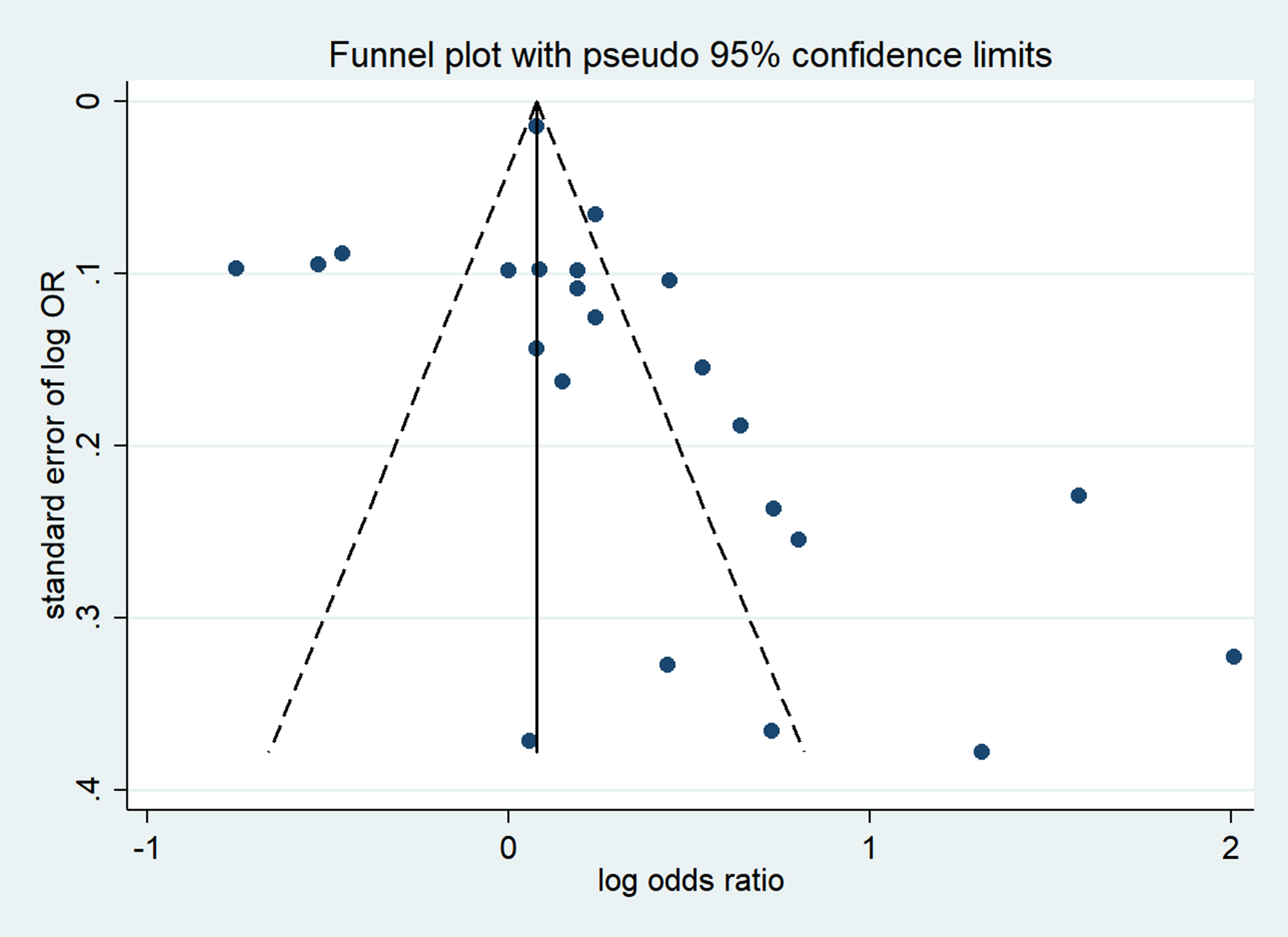

Supplement: Supplementary Figure 3 — Funnel plot of mild cognitive impairment (MCI). [file Image_3.TIF]

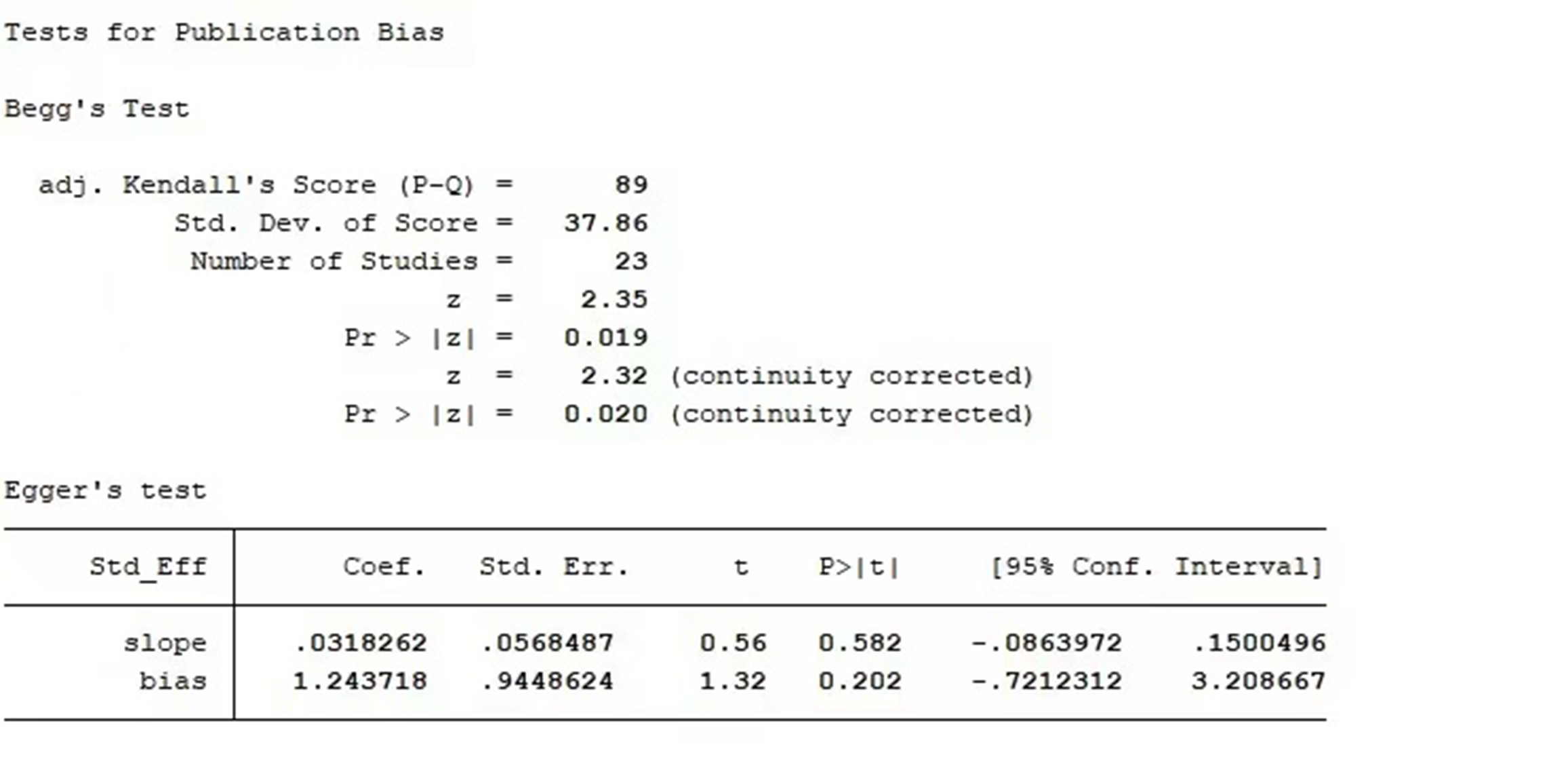

Supplement: Supplementary Figure 4 — Egger's test of mild cognitive impairment (MCI). [file Image_4.TIF]
