# Supplementary material for: Association between sedentary behavior and risk of cognitive decline or mild cognitive impairment among the elderly: a systematic review and meta-analysis
Source: Front Neurosci. 2023 Aug 4;17:1221990. doi: 10.3389/fnins.2023.1221990 (PMC10436513; doi:10.3389/fnins.2023.1221990)
Supplement: Supplementary Table 1 — Quality assessment for inclusion in cohort studies. [file Table_2.DOCX]

**Supplementary Table 1. Quality assessment for inclusion in cohort studies.**

| **Study Year** | **Selection** | | | | **Comparability** | **Exposure** | | | **Total score** |
| --- | --- | --- | --- | --- | --- | --- | --- | --- | --- |
|  | **Representativeness of the exposed cohort** | **Selection of the non exposed cohort** | **Ascertainment of exposure** | **Demonstration that outcome of interest was not present at start of study** | **Comparability of cohorts on the basis of the design or analysis** | **Assessment of outcome** | **Was follow up long enough for outcomes to occur** | **Adequacy of follow up of cohorts** |  |
| Ku 2017 | 1 | 1 | 1 | 1 | 2 | 1 | 0 | 1 | 8 |
| Ferreira 2010 | 1 | 1 | 1 | 1 | 2 | 1 | 0 | 0 | 7 |
| Lee 2013 | 1 | 1 | 1 | 1 | 2 | 1 | 1 | 1 | 9 |
| Song 2022 | 1 | 1 | 1 | 1 | 2 | 1 | 1 | 0 | 8 |
| Brunner 2016 | 1 | 1 | 1 | 0 | 2 | 1 | 1 | 1 | 8 |
| Ku 2017 | 1 | 1 | 1 | 1 | 1 | 1 | 0 | 1 | 7 |
