# Supplementary material for: Association between sedentary behavior and risk of cognitive decline or mild cognitive impairment among the elderly: a systematic review and meta-analysis
Source: Front Neurosci. 2023 Aug 4;17:1221990. doi: 10.3389/fnins.2023.1221990 (PMC10436513; doi:10.3389/fnins.2023.1221990)
Supplement: Supplementary Table 2 — Quality assessment for inclusion in cross-sectional studies. [file Table_3.DOCX]

**Supplementary Table 2. Quality assessment for inclusion in cross-sectional studies.**

| **Study Year** | **1** | **2** | **3** | **4** | **5** | **6** | **7** | **8** | **9** | **10** | **11** | **Total score** |
| --- | --- | --- | --- | --- | --- | --- | --- | --- | --- | --- | --- | --- |
| Gomes 2017 | Yes | Yes | Yes | Yes | Unclear | Yes | Yes | Yes | Yes | Yes | Unclear | 9 |
| Nemoto 2018 | Yes | Yes | Yes | Yes | Unclear | Yes | Yes | Yes | Yes | Yes | Unclear | 9 |
| García-Hermoso 2018 | Yes | Yes | Yes | Yes | Unclear | Yes | Yes | Yes | Unclear | Yes | Unclear | 8 |
| Dogra 2012 | Yes | Yes | Yes | Yes | Unclear | Yes | Yes | Yes | Unclear | Yes | Unclear | 8 |
| Vancampfort 2018 | Yes | Yes | Yes | Yes | Unclear | Yes | Yes | Yes | Yes | Yes | Unclear | 9 |
| Lara 2016 | Yes | Yes | Yes | Yes | Unclear | Yes | Yes | Yes | Yes | Yes | Unclear | 9 |
| Cui 2021 | Yes | Yes | Yes | Yes | Unclear | Yes | Yes | Yes | Unclear | Yes | Unclear | 8 |
| Du 2022 | Yes | Yes | Yes | Yes | Unclear | Yes | Yes | Yes | Unclear | Yes | Unclear | 8 |
| Gillum2015 | Yes | Unclear | Yes | Yes | Unclear | Yes | Unclear | Yes | Unclear | Yes | Unclear | 6 |
| Jung 2020 | Yes | Yes | Yes | Yes | Unclear | Yes | Yes | Yes | Yes | Yes | Unclear | 9 |
| Martínez-Sanguinetti 2019 | Yes | Yes | Yes | Yes | Unclear | Yes | Yes | Yes | Yes | Yes | Unclear | 9 |
| Paulo 2016 | Yes | Yes | Yes | Yes | Unclear | Yes | Yes | Yes | Yes | Yes | Unclear | 9 |
| Poblete-Valderrama 2019 | Yes | Yes | Yes | Yes | Unclear | Yes | Unclear | Yes | Unclear | Yes | Unclear | 7 |

1. Whether the sources of data (survey, literature review) are identified; 2. Whether inclusion and exclusion criteria for exposed and non-exposed groups (cases and controls) are listed or reference is made to previous publications; 3. Whether the time stage of patient identification is given; 4. If not from the population, whether the subjects are continuous; 5. Whether the evaluator's subjective factors cover up other aspects of the research object; 6. Describes any assessment undertaken to ensure quality (e.g. testing/retesting of primary outcome indicators); 7. The reasons for excluding any patients in the analysis are explained; 8. Measures to evaluate and/or control confounding factors are described; 9. Where possible, explain how the lost data is handled in the analysis; 10. The response rate of patients and the integrity of data collection were summarized; 11. If follow-up is available, identify the expected percentage of patients with incomplete data or follow-up results.
